# Melatonin Distribution Reveals Clues to Its Biological Significance in Basal Metazoans

**DOI:** 10.1371/journal.pone.0052266

**Published:** 2012-12-26

**Authors:** Modi Roopin, Oren Levy

**Affiliations:** The Mina & Everard Goodman Faculty of Life Sciences, Bar-Ilan University, Ramat-Gan, Israel; Karlsruhe Institute of Technology, Germany

## Abstract

Although nearly ubiquitous in nature, the precise biological significance of endogenous melatonin is poorly understood in phylogenetically basal taxa. In the present work, we describe insights into the functional role of melatonin at the most “basal” level of metazoan evolution. Hitherto unknown morphological determinants of melatonin distribution were evaluated in *Nematostella vectensis* by detecting melatonin immunoreactivity and examining the spatial gene expression patterns of putative melatonin biosynthetic and receptor elements that are located at opposing ends of the melatonin signaling pathway. Immuno-melatonin profiling indicated an elaborate interaction with reproductive tissues, reinforcing previous conjectures of a melatonin-responsive component in anthozoan reproduction. In situ hybridization (ISH) to putative melatonin receptor elements highlighted the possibility that the bioregulatory effects of melatonin in anthozoan reproduction may be mediated by interactions with membrane receptors, as in higher vertebrates. Another intriguing finding of the present study pertains to the prevalence of melatonin in centralized nervous structures. This pattern may be of great significance given that it 1) identifies an ancestral association between melatonin and key neuronal components and 2) potentially implies that certain effects of melatonin in basal species may be spread widely by regionalized nerve centers.

## Introduction

Melatonin is one of the phylogenetically oldest biological molecules in nature. An exceptional spectrum of action for this pleiotropic agent has been uncovered in a nearly ubiquitous range of organisms (e.g., [1]–[6]); however, the principle significance of melatonin in many non-vertebrate species remains uncertain. This apparent knowledge gap is largely because insufficient effort has been exerted in the investigation and characterization of the specific role of melatonin in many of the numerous non-vertebrate organisms that have been demonstrated to possess endogenous melatonin activity. Investigations in such species have primarily focused on either the classical vertebrate function of melatonin as a transducer of photoperiodic information, or more recently on its putative role as an antioxidative protectant (e.g., [2]–[4], [7]–[9]). Of the well-established functions in vertebrates, the involvement of melatonin in the detoxification of destructive radicals, is neither species- nor cell-specific (e.g., [10], [11]) and has therefore been hypothesized to represent the primary role of melatonin in living organisms [9]. Nonetheless, the benefits of melatonin as an antioxidant have only been demonstrated in a small number of non-vertebrate species to date, primarily dinoflagellates (e.g., [12], [13]). The extent to which antioxidant activity represents the principal biological significance of melatonin in multicellular heterotrophic non-vertebrates remains largely theoretical. Another fundamental function of melatonin in vertebrates relates to its key role as a photoperiodic synchronizer of biological processes (reviewed in [14], [15]). However, while hypotheses concerning an early evolutionary photoperiodic role of melatonin in basal species have been historically appealing, the combined findings from various non-vertebrate species suggest potentially divergent patterns among different organisms and even different organs [2]–[5].

Anthozoa represent the most basal class within the Cnidaria [16]–[18], which is the simplest animal phylum at the level of tissue organization. Thus, the role of melatonin in anthozoans may represent an informative and restricted subset of early-evolved functionality at the base of metazoan evolution. Nonetheless, only isolated studies of melatonin exist for this phylogeny level. The findings from previous studies conflict regarding the daily pattern of melatonin changes. For example, the sea pen *R. koellikeri* exhibits 24-hour arhythmicity in melatonin levels [19], whereas the sea anemone *A. equina* exhibits significant day-night rhythms [20]. However, all studies generally agree on the potential involvement of this indoleamine in reproductive processes. In the sea pen *R. koellikeri*, the involvement of melatonin in reproduction was strongly implied by three parameters: 1) the correspondence of seasonal alterations in melatonin levels with the first stages of sexual maturation, 2) substantially higher melatonin levels in the gamete-bearing colonial mass, and 3) a potentially neuronal distribution of melatonin immunoreactivity in the endodermal filaments that are wrapped around gametes [19]. Given that anthozoans are the lowest animal group with a nervous system [21]–[24], any melatonin activity that involves neuronal interactions in these species is intriguing. Neuronal melatonin may represent a primordial association between melatonin and the nervous system and may also provide a novel understanding of the function of melatonin during early metazoan evolution. However, comparative evaluations of melatonin’s neural interactions across additional anthozoans and other basal species are required to fully assess the significance of this potential relationship at the base of metazoan evolution. In vertebrates, the neuro-regenerative and neuro-protective effects of melatonin are known to involve both receptor-coupled and receptor-independent processes (reviewed in [25]). Although receptor-independent neuro-protective effects of melatonin can be hypothesized to occur in basal metazoan species as part of the general antioxidant role of this molecule, even the most basic mechanistic understanding of the relationship between melatonin and the ancient neural network in anthozoans is lacking.

The present study reveals for the first time the distribution of melatonin in whole specimens of the starlet sea anemone *Nematostella vectensis*, which is a key anthozoan model organism. Although the distribution patterns of melatonin in *Nematostella* support previous conjectures concerning its role in reproduction [19], [20], elevated melatonin was predominantly observed in distinct neural locations within *Nematostella,* suggesting a dominant neuronal role in its mechanism of action. Moreover, spatial expression profiles of putative genes for melatonin biosynthetic and receptor elements [26], which were hitherto unstudied, supported the neural distribution of this molecule and suggested that the action of melatonin in anthozoans may be modulated through receptor interactions, as in vertebrates.

## Materials and Methods

### Nematostella Culture

*Nematostella vectensis* were cultured as previously described [27]. Adult polyps were fed at least four times a week with *Artemia nauplii,* and water changes were conducted after feeding. The adult polyps were regularly spawned following a previously established protocol [27].

### Melatonin Extraction

Melatonin was extracted from adult polyps as described in Roopin and Levy [20]. For further purification of extracts, 1 mL from each extract was processed on a solid phase extraction (SPE) cartridge (Oasis HLB, Waters), according to the manufacturer’s instructions. The purified extract was evaporated to dryness in a vacuum concentrator (Heto-Holten, Denmark) and re-dissolved in 100 µL of the mobile phase (see below) for liquid chromatography analyses.

### HPLC

The detection of melatonin in *N. vectensis* samples was initially performed on a Hitachi high precision liquid chromatograph (HPLC) system (LaChrom Elite, IL, USA) that was equipped with a fluorescence detector (Hitachi L-2485). The separation was performed using a Phenomenex Luna RP C18(2) 5 µm (100×4.6 mm) cartridge at 30°C. The mobile phase consisted of a mixture of 0.1% (v/v) formic acid in acetonitrile:water 17∶ 83 (v/v) and was delivered isocratically at a flow rate of 1 mL min^−1^. A volume of 15 µL from each extracted, SPE-purified sample was injected into the HPLC system. The fluorescence intensity was recorded at λ_em_ = 341 nm with excitation at λ_ex_ = 297 nm to detect the melatonin peak. Double distilled water (DDW) and freshly prepared culture medium were tested as controls. Calibration curves were preformed using freshly prepared melatonin standards prior to the analyses. The EZChrom Elite™ Chromatography Data System Software was used for integrations.

### ESI–MS–MS Detection

To further confirm our HPLC-based identification of melatonin in *N. vectensis*, representative samples (N = 3) were subjected to liquid chromatography-electrospray ionization-tandem mass spectrometry (LC/ESI/MS/MS) analysis. The LC-MS system was equipped with an Agilent 1100 series HPLC (Palo Alto, CA) with a G1314A UV detector and a Luna RP C18(2) 5 µm (100×4.6 mm) column (Phenomenex, CA, USA). The conditions that were used for LC-MS were the same as those described for HPLC, except for a slightly lower flow rate (0.85 mL min^−1^). A volume of 10 µL of each sample was injected into the system. The mass spectra were obtained using a Bruker MS Esquire 3000 Plus (Billerica, MA, USA) that was equipped with an electrospray source and an ion trap detector that was operated in positive electrospray ionization mode (ESI+). Full scan spectra were acquired in continuum mode over the mass range of 150–300 u at a scan rate of 500 u/s. Moreover, protonated molecules ([M+H]^+^) in the sample were subjected to collision-induced dissociation (CID) in the octapole collision cell, using helium as the collision gas, with typical collision energies of 25 eV. Selected ion recording (SIR) data were acquired by sequentially monitoring quasi-molecular ions of [melatonin+H]^+^ at an m/z of 233. The determination of melatonin in the unknowns was thereafter performed in multiple reaction monitoring (MRM) mode based on the fragmentation patterns of a melatonin standard (e.g., [28]–[30]). The m/z transitions from 233 to 174 and from 233 to 216 were used for MRM detection. Prior to the analyses of the *N. vectensis* samples, confirmative tests of the system included control runs using DDW and the characterization of melatonin fragmentation on freshly prepared melatonin standards. Agilent ChemStation software was used for data acquisition and peak area integration. The instrument control and data analyses were conducted with Bruker Daltonics Data Analysis software version 3.0.

### Immunochemistry

*N. vectensis* specimens at pre-adult stages were fixed in fresh ice-cold 3.7% formaldehyde that contained 0.2% glutaraldehyde in 1/3 × seawater for 90 sec. The specimens were then post-fixed in 3.7% formaldehyde in 1/3 ×seawater at 4°C for 1 h. The adult polyps were relaxed in 7% MgCl_2_ for 10 min and fixed in fresh ice-cold 3.7% formaldehyde that contained 0.2% glutaraldehyde in 1/3 × seawater for 10 min prior to post-fixation in 3.7% formaldehyde in 1/3 × seawater at 4°C for 2 h. The fixed specimens were washed in PTw (phosphate-buffered saline, 0.1% Tween-20) and transferred to 100% methanol for storage at −20°C. A subset of the adult polyps were paraffin-embedded, and 6 µm sections were prepared.

For the immunostaining, the samples were thoroughly washed five times for 30 min in PTw and blocked in 10% normal donkey serum (Jackson ImmunoResearch Laboratories, PA, USA, catalog no. 017-000-121) in PTw (blocking solution) for 1 h. The samples were then incubated overnight at 4°C with sheep anti-melatonin serum that was diluted 1∶500 in blocking solution (Stockgrand, Ltd., Guildford, U.K.; Batch AB/S/01). Following the incubation, the samples were subjected to five additional 30 min washes with PTw at room temperature, re-blocked for 30 min, and incubated overnight at 4°C with Rhodamine Red-X-AffiniPure Donkey Anti-Sheep IgG (Jackson ImmunoResearch Laboratories, PA, USA) that was diluted 1∶200 in blocking solution. Lastly, the samples were rapidly washed three times, washed five additional times for 30 min with PBS, and mounted in Fluoromount G® (Dako Industries). For the controls, the primary antibody was excluded from the above procedure, or the primary antibody was neutralized by preadsorption with melatonin at a final concentration of 10^−3^ M. For the double-labeling of melatonin and FMRFamide, which is a well characterized neuronal marker in invertebrates, the samples were incubated overnight at 4°C with a mixture of the melatonin antiserum and rabbit anti-FMRFamide serum (Peninsula Laboratories, Europe Ltd., St. Helens, UK, IHC 8755) that was diluted 1∶300 in blocking solution. The FMRFamide was detected using DyLight 649 AffiniPure Donkey Anti-Rabbit IgG (Jackson ImmunoResearch Laboratories, PA, USA), which was diluted 1∶200 in blocking solution.

The staining of sections and whole mounts was viewed and photographed using an AxioImager Z1 epifluorescence microscope that was equipped with an AxioCam CCD camera (Carl Zeiss, Jena, Germany) and a confocal laser scanning microscope (Zeiss LSM 510 META). All of the images were obtained under constant exposure conditions. The levels of the individual fluorescent channels were adjusted in Adobe Photoshop for certain images; however, the relative relationship of the pixels within these channels was not altered.

### In situ Hybridization

The spatial expression patterns of two representative Joint Genome Institute (JGI)-predicted gene orthologs for Hydroxyindolamine-O-methyltransferase (*HIOMT*, protein ID’s 91623, 123672, [26]) and two representative melatonin receptor orthologs (protein ID’s 209463, 13917, [26]) were evaluated using in situ hybridization (ISH). Digoxigenin (DIG)-labeled riboprobes (0.6–1 kb) for these four sequences were synthesized using the DIG RNA Labeling Kit (SP6/T7; Roche, Mannheim, Germany) and hybridized to paraffin-embedded *Nematostella* tissue sections. Prior to the hybridization, the tissue sections were subjected to a 15–30 min treatment with PTw that contained 10 µg mL^−1^ Proteinase K. This treatment was stopped by incubation in 4 mg mL^−1^ glycine in PTw for 10 min. The tissue sections were then incubated for 5 min in 100 mM triethanolamine (TEA), 5 min in 100 mM TEA/0.25% acetic anhydride and 5 min in 100 mM TEA/0.5% acetic anhydride. After two thorough washes in PTw, the polyps were fixed in 4% paraformaldehyde for 20 min, washed five times for 5 min in PTw, and pre-hybridized in a solution that contained 50% formamide, 5 × SSC, 200 µg mL^−1^ yeast RNA, 0.1% Chaps, 1 × Denhardt’s, 100 µg mL^−1^ heparin, and 0.1% Tween20 for 2 h at 65°C. After adding heat-denatured DIG-labeled riboprobes (0.1 ng µL^−1^), the samples were hybridized for an additional 18–24 h at 65°C. This step was followed by several washes in PTw with decreasing concentrations of hybridization solution. The hybridized tissues were then blocked for 2 h in blocking solution [1% blocking reagent (Roche) in PTw, 0.02% sodium acid]. Probe detection was achieved by incubation of the DIG-labeled samples with an anti-digoxigenin alkaline phosphatase (AP)-conjugated antibody (Roche, Basel, Switzerland) at 1∶3000 in blocking solution overnight (4°C). The following morning, after incubating for 5 min in both NTMT (100 mM NaCl, 100 mM Tris pH 9.5, 50 mM MgCl2, 0.1% Tween20) and 1 mM Levamisole/NTMT, the staining was detected using the substrate nitroblue tetrazolium-5-bromo-4-chloro-3-indoyl phosphate, p-toludine salt (NBT/BCIP, Roche 650 solution). The duration of the colorimetric reaction with the substrate was varied to reveal different expression levels. For the control samples, we either excluded the application of a labeled probe, used a complementary probe (non-HIOMT, ‘sense’), or excluded the application of the anti-Dig/AP antibody in the above procedure. The labeled specimens were viewed and photographed using an AxioImager Z1 epifluorescence microscope that was equipped with an AxioCam CCD camera (Carl Zeiss, Jena, Germany).

## Results

**Figure 1:**
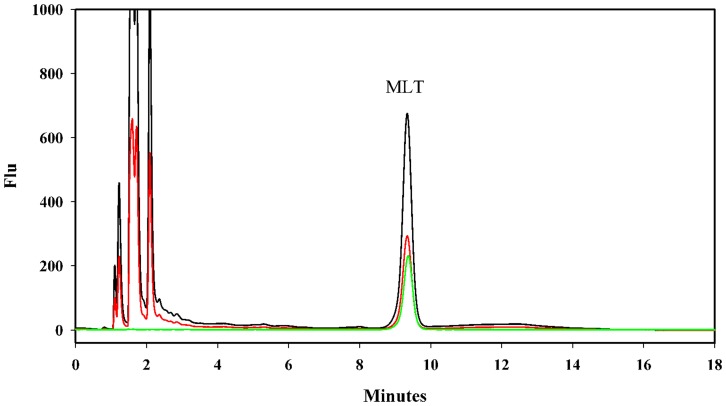
The detection of melatonin by HPLC. Overlaid chromatograms of a melatonin standard (green, 1×10^−8^ gr mL^−1^) and a sea anemone tissue extract (*N. vectensis*) with (black) and without (red) melatonin enrichment (1×10^−8^ gr mL^−1^). Chromatographic separation of tissue extracts was conducted based on fluorimetric detection (λex = 280 nm and λem = 345 nm). The mobile phase consisted of a mixture of 0.1% (v/v) formic acid in acetonitrile:water 17∶81 (v/v), which was delivered isocratically at a flow-rate of 1 mL min^−1^. The detailed conditions are described in the Materials and Methods.

**Figure 2:**
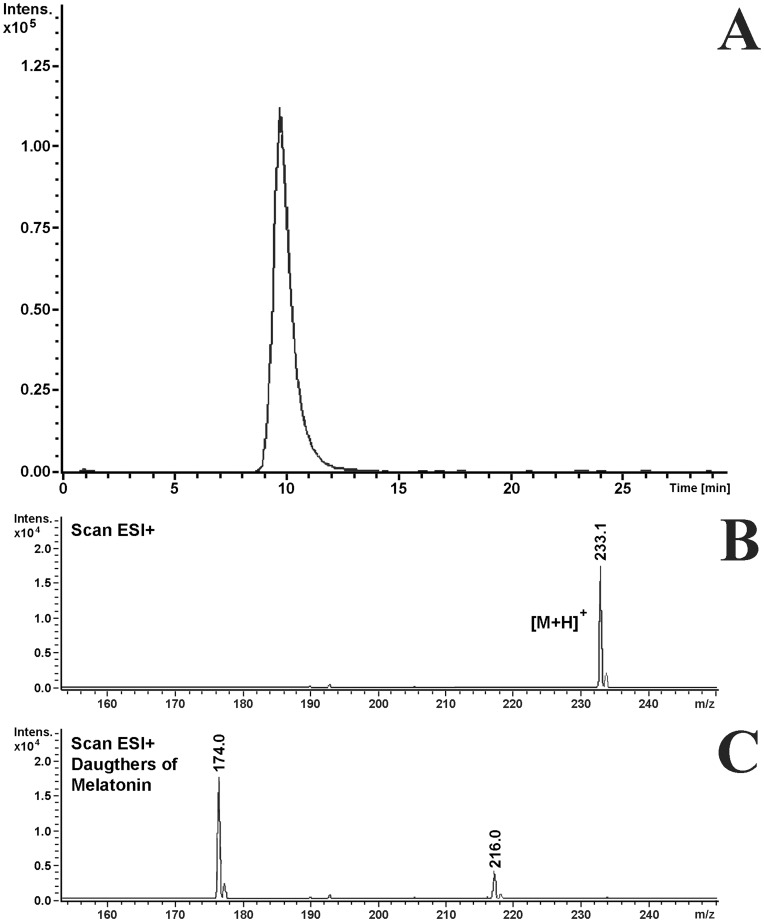
The detection of melatonin by HPLC/ESI/MS-MS. The mass chromatogram that was obtained using the MRM detection mode (A), and positive-ion scan spectra of the melatonin parent (B) and daughter ions (C) in a representative sample of *N. vectensis*. Experimental conditions: C18 column; a mobile phase acetonitrile:water 17∶83 (v/v) that contained 0.1% formic acid and which was delivered isocratically; flow rate: 0.85 mL min^−1^; temperature: 30°C; injection volume: 10 µL; electrospray ionization in positive mode; mass spectrometric detection in multiple reaction monitoring mode (selected transitions: 233 to 216 and 233 to 174). The detailed conditions are described in the Materials and Methods.

The fluorescent HPLC analysis of melatonin-spiked samples and melatonin standards allowed for the identification of a characteristic peak that corresponded to melatonin in *N. vectensis* tissue extracts (retention time: 9.4 min; Fig. 1). The addition of authentic melatonin to samples prior to extraction increased the intensity of this putative melatonin peak. LC/ESI/MS/MS unequivocally confirmed HPLC-based identifications of melatonin and excluded possible co-elution of other compounds with the same retention time. Tandem mass spectrometry (MS/MS), which was used here, is an extremely reliable technique in which a parent ion is mass-selected and induced to fragment into structurally significant fragmentation signatures [28]–[30]. Melatonin is more likely to gain than to lose a proton under acidic electrospray ionization conditions; therefore, the positive electrospray ionization mode was the detection method that was chosen for this analysis. A full scan spectra revealed that melatonin produced a quasi-molecular ion [M+ H]+ at m/z 233 (Fig. 2). Daughter spectra may be obtained by selecting these quasi-molecular ions as parent ions. The prominent daughter ions had m/z values of 174 and 216 (Fig. 2), corresponding to losses of 17 and 59 u, respectively [28], [29]. The purification of extracts with SPE and the use of a separation column further excluded interference from other endogenous compounds in the sample that may otherwise be co-eluted with melatonin. This allowed for reliable mass detection using the MRM mode. The presence of melatonin was confirmed in all of the *N. vectensis* samples that were subjected to this analysis (N = 3). No melatonin was identified in the DDW or the freshly prepared culture medium controls.

**Figure 3:**
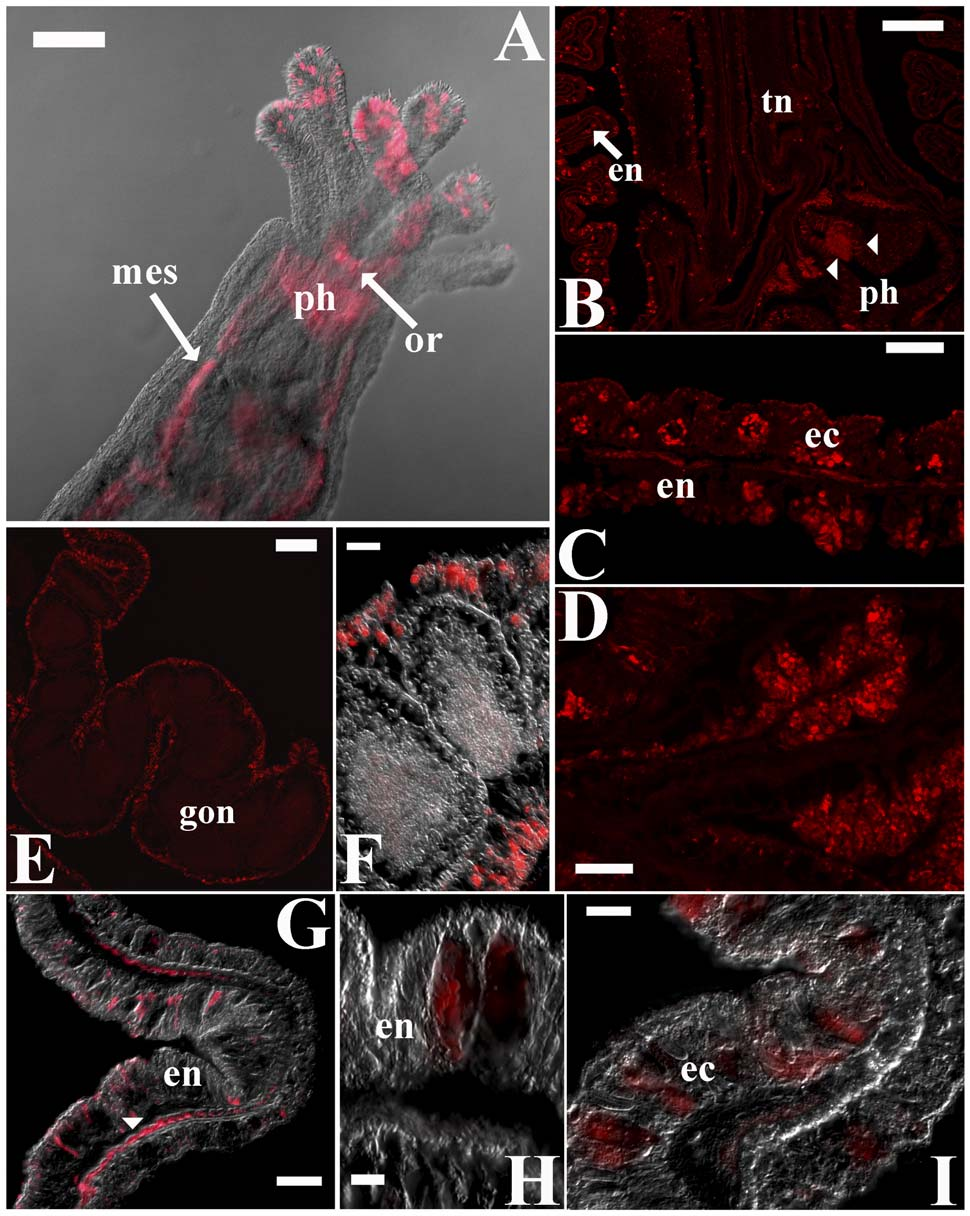
The distribution of melatonin immunoreactivity in *Nematostella vectensis* polyps. (A) Overlaid fluorescent and differential interference contrast (DIC) micrographs of a young adult polyp that was stained with a melatonin-specific antibody (red). Melatonin accumulation is noticeable in the tentacle tips (tn), mesenteries (mes) and actinopharynx (ph). Oral ring (or). (B–C) Confocal micrographs of melatonin immunoreactivity in *Nematostella* sections. Melatonin appears to be unevenly distributed throughout the layers (arrow) of the hypostomal body wall (retracted polyp), implying layer-specific differences in melatonin sources/targets (B, C). (D) Increased magnification of extensive melatonin accumulation in the outer surfaces of the endothelium in the actinopharynx folds (arrowheads in B). (E) The punctate immunoreactive pattern lining the external surfaces of the gonads suggests a reproductive role for melatonin. (F) Uniform melatonin immunoreactivity was detected in gonadal endodermal cells. (G-I) Melatonin immunoreactivity was observed within both endodermal and ectodermal cells in the body wall; the endodermal signals were generally stronger. Occasionally, melatonin accumulated at the base of the endoderm, implying an interaction with epithelial muscular cells (arrowhead). Scale bars: B = 200 µm; A, E = 100 µm; C, D, F, G = 20 µm; I = 10 µm; H = 5 µm.

The immuno-labeling of adult polyps indicated a highly diffuse distribution of melatonin. However, despite this widespread pattern (Fig. 3), stronger melatonin immunoreactive signals were consistently localized to specific areas, such as the tentacle tips, the actinopharynx and the septal filaments (Fig. 3A). The staining of longitudinal sea anemone sections also revealed cellular deposits of melatonin in the body wall areas. For example, the hypostome and its adjacent bi-layered body wall areas consistently displayed intense melatonin signals (Fig. 3B, C) that were heterogeneously distributed among the cells. These signals were observed in only a subset of the epithelial cells in each layer, forming irregular, potentially layer-specific patterns. Conversely, intense punctate melatonin signals appeared to originate from all of the endodermal gonad cells in the reproductive tissues, without exception (Fig. 3E, F).

**Figure 4:**
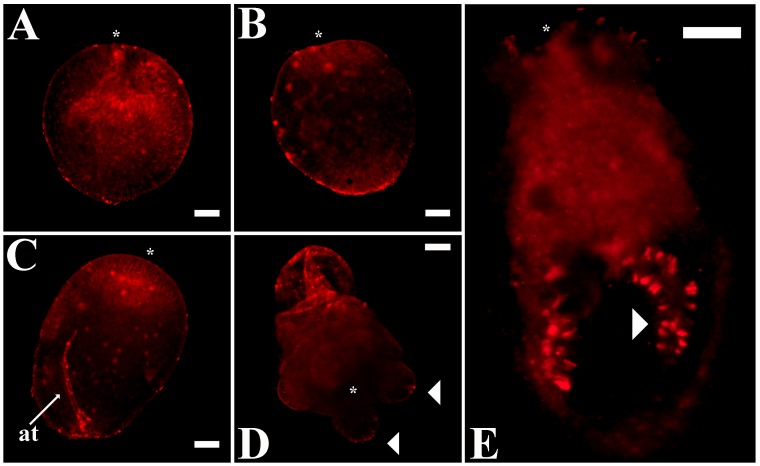
Melatonin expression patterns in *Nematostella vectensis* developmental stages. Confocal sections of whole mounts indicated that melatonin is enriched near the area of invagination in the early gastrula stage (A) and at the oral and aboral poles during the late gastrula stage of the forming planulae (B). In swimming planulae (older larvae with an actinopharynx and developing mesenteries), melatonin exhibited a definite early preference for the actinopharynx and the apical tuft (at), both of which are highly neuralized components of the developing nerve net (C). By the 4-tentacle primary polyp stage (D, E), this preference extends to include other neural areas, such as the tentacle (tn) tips (arrowheads) and mesenteries (mes, arrowhead). The concentration of melatonin in the developed mesenteries is very clear in (E). The asterisks denote the oral poles. Scale bars: A-E = 50 µm.

To clarify whether differences in melatonin distribution can be identified across early development, we evaluated melatonin immunoreactivity at different stages of *Nematostella* morphogenesis (Fig. 4). Comparable patterns were visible from the early gastrula stage through to the polyp stage and indicated an early preference for melatonin to localize to the actinopharynx and apical tuft areas, both of which are highly neuralized components of the developing nerve net [31]. Strong melatonin signals in the mesenteries were evident only at later stages of polyp development (Fig. 4D, E).

**Figure 5:**
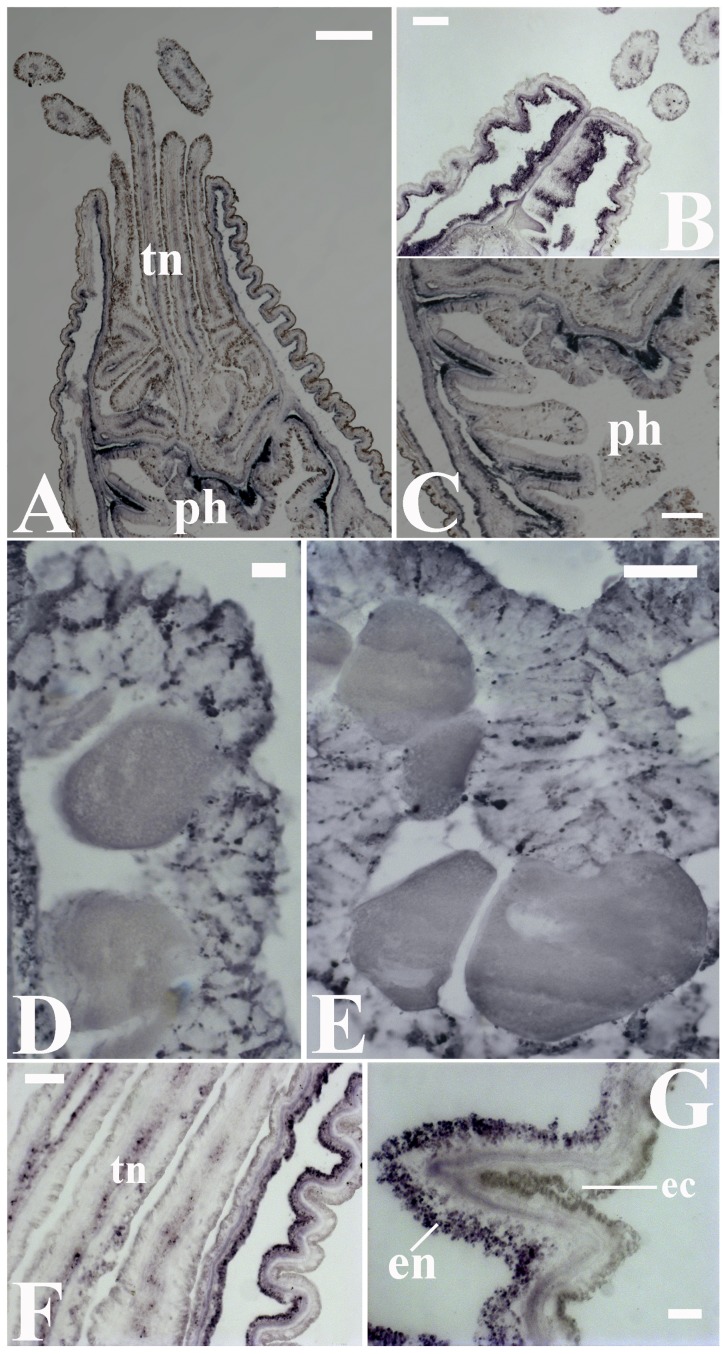
The expression pattern of hydroxyindole-O-methyltransferase (*HIOMT*) mRNA in *Nematostella vectensis*. Two representative *HIOMT* orthologs were evaluated using in situ hybridization (ISH) with specific probes. (A–C) The *HIOMT* expression pattern was similar between orthologs and indicated that melatonin is predominantly produced in the circumference of the actinopharynx (ph). High *HIOMT* expression levels were also evident in the endodermal layer of the hypostome (retracted individual, B). A higher magnification image of the outer surface of the endothelium in the actinopharynx folds (C). (D, E) Substantial *HIOMT* expression in reproductive tissues suggested that the considerable level of the melatonin that is observed in these tissues (see Fig. 1E–F) is locally produced. A uniform *HIOMT* expression pattern was observed among the cells. (F, G) In the body wall, predominantly endodermal *HIOMT* expression was observed throughout the apical end of the anemone and was uniformly distributed throughout the cells. This pattern differed from the pattern of melatonin immunoreactivity (see Fig. 1B–C). Note that melatonin production occurs also in tentacles (tn), albeit at lower levels. Endoderm (en), ectoderm (ec). Scale bars: A = 200 µm; B, C = 100 µm; E, F = 50 µm; D, G = 20 µm.

*HIOMT* (also referred to as acetyl serotonin methyl transferase, *ASMT*) is a key enzyme that catalyzes the conversion of N-acetylserotonin to melatonin in the final step of the melatonin pathway in tryptophan metabolism [32], [33]. The evaluation of two putative *Nematostella* orthologs of this enzyme [26] indicated analogous gene expression patterns that were highly correlated with the spatial distribution of melatonin immunoreactivity. Significantly increased putative *Nematostella HIOMT* expression at the hypostome and actinopharynx re-emphasized the potential importance of these structures as major sources of extensive melatonin production (Fig. 5A–C). Moreover, substantial levels of putative-*HIOMT* mRNA were detected in the endodermal cells of gametogenic mesenteries (Fig. 5D, E). The expression of putative *HIOMT* in the body wall provided yet another informative insight into melatonin production, as this enzyme was expressed at higher levels in the endoderm layer (Fig. 5F, G).

**Figure 6:**
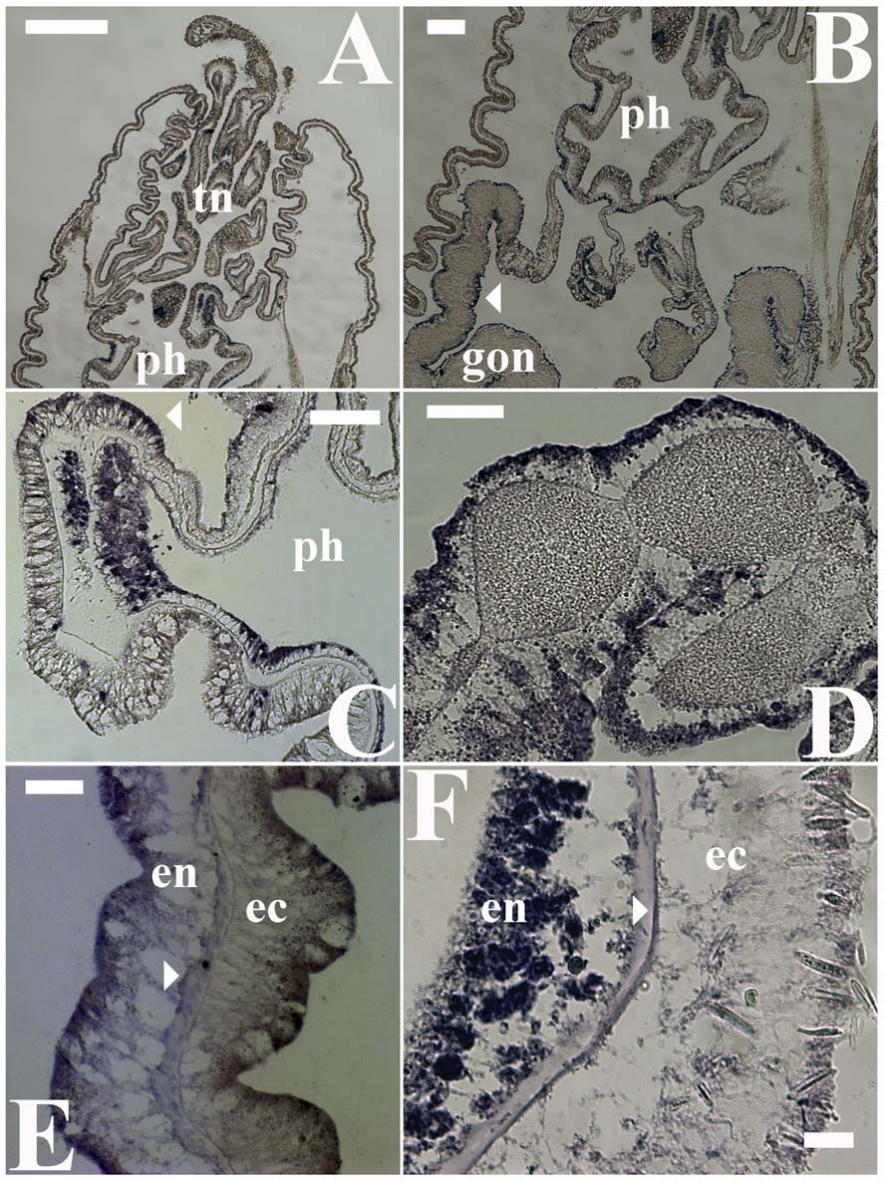
Gene expression patterns of putative melatonin receptors in *Nematostella vectensis*. Two representative melatonin receptor orthologs were evaluated using in situ hybridization (ISH) with specific probes. (A, B) A similar expression pattern between orthologs in adult polyps was observed and indicated that the mRNA for the putative receptor was predominantly expressed in the circumference of the actinopharynx (ph) and at substantially lower levels in the hypostome. (C) Higher magnification of the actinopharynx area indicated that the melatonin receptors are also expressed in the inner epithelium (arrowhead); albeit at lower levels (D) Substantial melatonin receptor expression was evident in fertile gonads (peripheral surfaces of endodermal cells), implying a reproduction-related receptor-mediated mechanism of action for melatonin. (E, F) The expression of putative melatonin receptors in the body wall was predominantly characterized by an endodermal distribution but was also sporadically observed at lower levels in both ectodermal cells and in the epithelial muscular layer (arrowheads) at the base of the endoderm. Scale bars: A = 500 µm; B = 200 µm; C = 100 µm; D, E = 50 µm; F = 20 µm.

The expression patterns of two representative orthologs for putative *Nematostella* melatonin receptors [26] were similar but only partially corresponded to the expression profile of the putative *HIOMT*. The high density of these elements in the endodermal layer surrounding the actinopharynx suggested that melatonin’s functional relevance in this region may involve interaction with receptors. However, a generally lower density of putative melatonin receptors was observed in the hypostome area (Fig. 6A–C). Consistent with the observed patterns of putative *HIOMT* expression and melatonin immunoreactivity, considerable expression of the putative receptors was observed in fertile septal filaments (Fig. 6D). In the body wall, which was an area of potentially irregular melatonin immunoreactive signals, the expression of the putative receptors mRNA was primarily observed in the endoderm (Fig. 6E, F). However, lower mRNA expression was also evident in ectodermal cells and in endodermic epithelial-muscle cells near the mesoglea (Fig. 6E, F). Other key areas, such as the tentacle tips, exhibited relatively weak expression of the putative melatonin receptors, primarily at their endodermal side.

**Figure 7:**
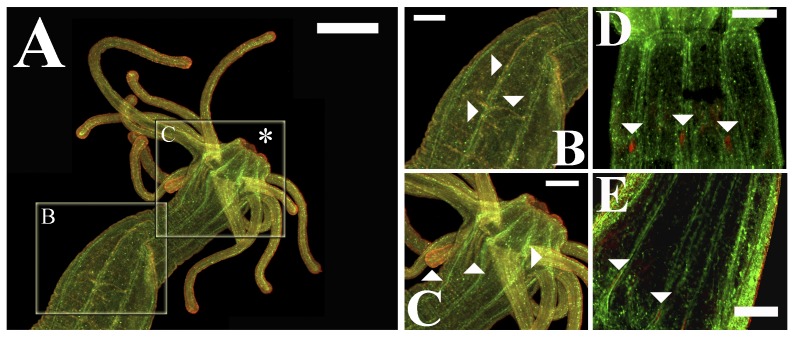
Confocal colocalization of melatonin and the *Nematostella vectensis* neural network in adult polyps. (A) The distribution of melatonin immunoreactivity (red) generally corresponded with RFamide-expressing neurons (green). (B) Neuro-melatonin interactions were implied by specific colocalization (orange) along both the major longitudinal fasciculated neurite tracts and the minor inter-crossing neuronal pathways (arrowheads). (C) Melatonin distribution also paralleled the neural tracts in other areas of the *N. vectensis* neural net, such as the tentacles and the mesenterial endomesodermal cells that were proximate to the actinopharynx (arrowheads). (D, E) Confocal sections in the pharyngeal nerve ring area indicated neuro-melatonin associations. The asterisk denotes the oral pole. Scale bars: A = 500 µm; B, C-E = 200 µm.

**Figure 8:**
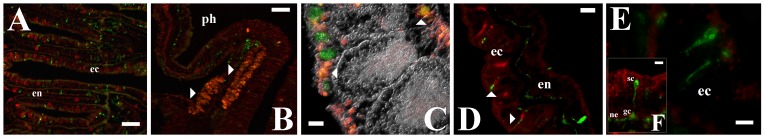
The colocalization of melatonin and the neural network in *Nematostella vectensis* sections. (A–C) An overlay of FMRFamide (green) and melatonin (red), revealing little colocalization (orange) in the hypostome (A), extensive colocalization in the actinopharynx (ph) (B) and sporadic neuro-melatonin interactions in a subset of the fertile gonad cells (arrowheads) (C). (D–F) Pervasive colocalization of melatonin and FMRFamide was observed in several neurons in the body wall (arrowheads) (D); however, sensory cells (sc), ganglion cells (gc) and basiepithelial neurites (ne) were generally not melatonin-positive despite being located in highly melatonin-enriched surroundings (E–F). Ectoderm (ec). Scale bars: A, B = 50 µm; C, D = 20 µm; E, F = 10 µm.

The colocalization of melatonin with FMRFamide, which is a known neuronal marker that is abundantly expressed in the anthozoan nervous system [34]–[36], was used to assess the probability of a functional relationship between melatonin and the anthozoan neural network. Despite certain regional differences, specific melatonin immunoreactive signals were observed along the central longitudinal neurite tracts, supporting the hypothesis that certain actions of melatonin may be mediated and/or propagated via central neuronal elements in *Nematostella* (Fig. 7). The colocalization of melatonin and FMRFamide was not restricted to the major neuronal tracts but was also distinguishable along the minor inter-crossing neuronal pathways (Fig. 7). Moreover, consistent melatonin signals were also visible in other highly innervated regions, such as the anemone tentacles and the pharyngeal nerve ring area (Fig. 7). Longitudinal sections provided greater resolution of the regional differences in the colocalization of melatonin and FMRFamide. For example, despite the widespread presence of melatonin in the hypostome, colocalization with FMRFamide in this region was low (Fig. 8A). In comparison, the actinopharynx area appeared to contain numerous putative neurons that were also distinctively melatonin-positive (Fig. 8B). Certain colocalization with FMRFamide-expressing cells was also apparent in reproductive filaments (Fig. 8C). In contrast, the body wall central nervous structures, such as the nerve plexus and sensory cells, were not convincingly melatonin-positive but were often surrounded by melatonin-rich cells (Fig. 8D–F). Thus, the distribution of melatonin not only strongly implies its association with the neural network but also suggests that the extent and nature of these interactions may vary between the highly innervated nerve centers and the peripheral tissues. The control immunohistochemistry and ISH analyses yielded no or very weak nonspecific background staining (Figs. S1, S2).

## Discussion

**Figure 9:**
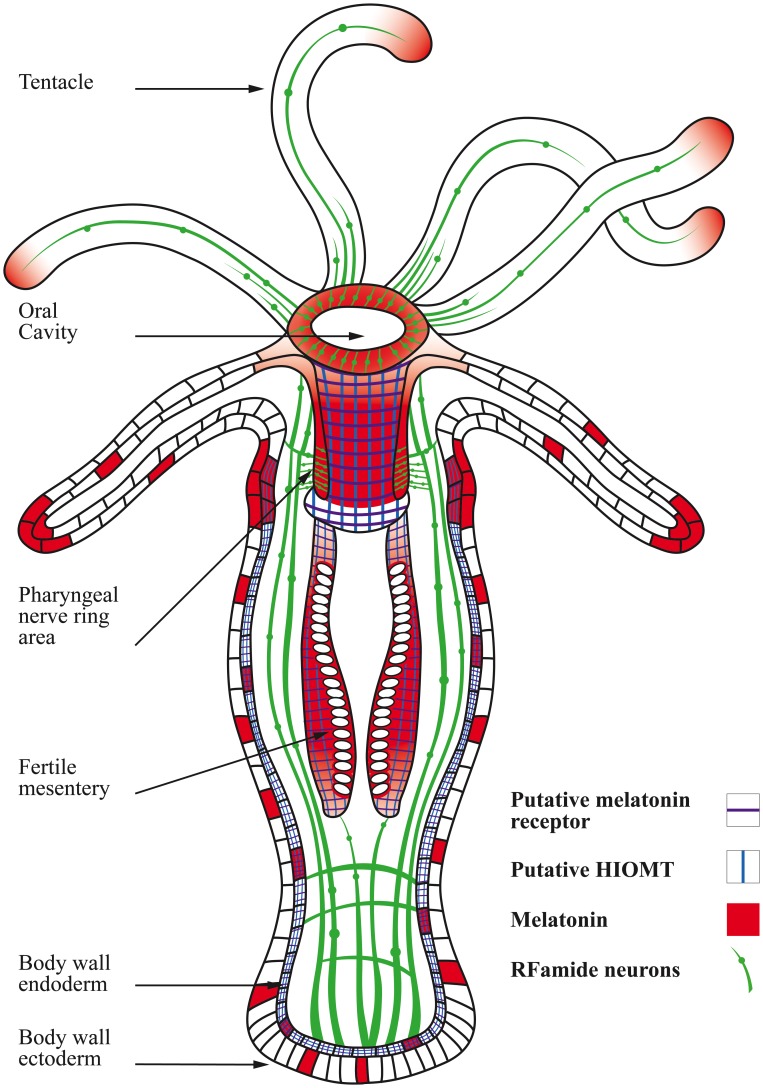
A schematic diagram of *Nematostella* illustrating the combinatorial expression of putative biosynthetic (*HIOMT*) and receptor gene elements overlaid with the distribution of melatonin immunoreactivity in different morphological features of the sea anemone. The neural architecture in *Nematostella* (as assayed by FMRFamide, which is a known neuro-marker in the anthozoan nervous system [34]–[36]), overlaps with melatonin immunoreactivity in several areas. *HIOMT* expression patterns are highly correlated with melatonin immunoreactivity in key neural areas and reproductive tissues, corresponding to abundant expression of putative melatonin receptors. Varying levels in the amount/abundance of melatonin or mRNA expression of the gene elements among different body regions are schematically represented in the diagram by varying color intensities or by the number of consecutive cells that indicate the presence of a particular element.

Anthozoans, as the earliest branching class of cnidarians [37], offer an exceptional opportunity to investigate biological questions that concern the functional significance of melatonin at the most “basal” level of metazoan evolution [38]. The sparse information that is currently available regarding the role of melatonin in anthozoans is perplexing. For example, diurnal melatonin arrhythmia has been correlated with rhythmic seasonality in the sea pansy *Renilla koellikeri* [19] while non-circadian diel periodicity was observed in the sea anemone *Actinia equina* [20]. These conflicting findings were recently reconciled by the hypothesis that environmental light, not endogenous circadian control, may be the major driving factor that underlies photoperiodic melatonin changes in anthozoans [20]. In the present work, hitherto uncharacterized immunohistochemical and ISH patterns offer a novel insight to melatonin’s actions in anthozoans by physically relating the distribution of this molecule to potential biosynthetic sources and putative receptor targets across different morphological features of *N. vectensis* (Fig. 9).

As the amphiphilicity of melatonin allows its easy passage across membrane barriers and diffusion through tissues, the widespread pattern of melatonin immunoreactivity that was observed in *N. vectensis* was unsurprising (e.g., [8], [39]). Regardless of this general pattern, intensified melatonin signals were consistently evident in reproductive tissues and areas of extensive neuronal innervation, highlighting a potential neuronal function of this molecule in anthozoans. The correspondence of putative *Nematostella HIOMT* expression with melatonin distribution not only confirmed the authenticity of immunoreactive-derived melatonin patterns but also added important support to the hypothesis that endogenous melatonin is synthesized from serotonin via tryptophan metabolism in basal metazoans. Additional evidence that links another element in this metabolic pathway to melatonin production was provided by a related study, in which external tryptophan treatment increased endogenous melatonin production in different sea anemone species than the one studied here [20]. It should be noted that, whereas O-methylation of N-acetylserotonin is likely catalyzed by a *HIOMT* homolog in *Nematostella*, arylalkylamine N-acetyltransferase (*AA-NAT*), which is a key melatonin biosynthetic enzyme that plays a central role in shaping the circadian pattern of melatonin synthesis in higher animals [40], has not been identified in the *N. vectensis* genome. Although *Nematostella* can evidently produce melatonin, presumably through the use of less specific N-acetyltransferases [3], [26], the apparent absence of *AA-NAT* in this species may underlie the non-circadian melatonin behavior in anthozoans [19], [20]. Moreover, measurably higher melatonin production in the hypostome and the actinopharynx, which is implied by elevated putative-*HIOMT* expression, implicates these regions as the chief contributors to any temporal pattern of global melatonin changes. In contrast to the above regions, a potential discrepancy between the intense, heterogeneous immuno-melatonin signals that were detected in the body wall (Fig. 3B, C, G) and the uniform, primarily endodermal, putative-*HIOMT* expression in the same tissue (Fig. 5F, G) implied that, although melatonin is primarily produced in endodermal cells, it is likely distributed to selected cellular targets in both layers. Alternatively, it is possible that *HIOMT* in the body wall may be engaged in other activities beside melatonin synthesis and that melatonin is actively produced only in cells that express a specific N-acetyltransferase. In any case, further study will be required to understand the exact mechanisms and the biological implications of these differences.

Melatonin plays a well-established role in the regulation of vertebrate seasonal reproductive physiology, particularly in mammalian seasonal breeders [41]–[43]. Seasonality is determined in these animals by measuring scotophase-dependent daily alterations in melatonin production, the duration of which is proportional to the daily dark period [43]–[45]. Although the site at which melatonin physically acts to regulate vertebrate seasonal reproductive capability has not been unequivocally identified [39], this effect is believed to be mediated via melatonin receptors, which are capable of modifying the cellular events that affect reproductive physiology (reviewed in [39], [46]). The involvement of melatonin in reproduction has been previously proposed to occur in several species in lower phyla, including sea anemones (reviewed in [20]); however, the molecular pathways for this potential action are unknown. Here, intense melatonin signals in *Nematostella*’s reproductive filaments were observed in neuron-like elements. Moreover, high expression of putative melatonin receptors was observed in fertile gonads. These patterns imply that, as in higher animals [47], the relationship between melatonin and gonadal growth in anthozoans may involve a mechanism in which melatonin levels modulate reproductive events by acting on neural elements that control gonadal development. Although the precise biological response of putative melatonin receptors in *Nematostella* requires additional study at the protein level, spatially relating the expression of these gene elements with melatonin distribution provides a strong and important basis for further investigation.

The principal action of melatonin in the nervous system of higher metazoans involves the transduction of daily and seasonal photoperiodic patterns into neuronal stimuli that entrain biological rhythms (reviewed in [48], [49]). The colocalization of melatonin with FMRFamide-expressing neurons in the *N. vectensis* nerve net suggests that neuro-melatonin communications may have evolved as a dominant feature of melatonin’s activity since the origin of metazoan evolution. The most interesting finding to support this hypothesis involves the intense melatonin immunoreactive signals that were distributed along the circumference of the actinopharynx (Figs. 3B, D), where distinct neuron populations form the pharyngeal nerve ring in *N. vectensis* [27]. The strong affinity of melatonin for the pharyngeal area was also evident during neurogenesis, further emphasizing the potential significance of this relationship. Moreover, the elevated expression of putative melatonin receptors suggests that melatonin is not only produced in high quantities around the pharyngeal region but also that G-protein-coupled transmembrane receptor (GPCRs) targets that potentially mediate its effects are abundantly available. Thus, our present ISH and immunocytochemistry data expand upon previous hypotheses that the actions of melatonin in anthozoans may be neural in nature, suggesting that a receptor-mediated interface may be involved in this pathway.

Although a complete understanding of the cnidarian nervous system has not been obtained, potentially centralized elements, such as circular nerve ring structures, are believed to represent complex features of these presumably simple systems (reviewed in [50]) and have been demonstrated to functionally coordinate a wide range of behavioral repertoires in certain species (e.g., [51], [52]). These structures have been proposed by several authors to be the cnidarian homolog of a central nervous system (CNS) [52], [53]. The dominant association of melatonin with the pharyngeal nerve ring in *N. vectensis* may imply that, in addition to having local effects in reproductive tissues [19], [20], this indoleamine molecule has a broader functional significance in basal metazoan biology by communicating with regionalized neural centers.

In summary, the morphological distribution patterns that are revealed in the present study provide an informative glance into two fundamental parameters that are likely to shape the functional activity of melatonin in *Nematostella*. First, the production of melatonin, which in this species appeared to be primarily endodermal, implicates the hypostome and actinopharynx as the major sources of melatonin. Moreover, the spatial correlation between melatonin and centralized nervous structures, such as the pharyngeal nerve ring, may be of great significance and implies a central role of melatonin in primordial organisms. Second, based on their spatial distribution and the relative expression of both coupled G-proteins and effector elements, melatonin receptors may modulate the action of melatonin, (reviewed in [54]). The pronounced expression of putative melatonin receptors in highly neuralized areas of the *N. vectensis* nerve net (e.g., the actinopharynx) and reproductive tissues highlights the possibility that receptor-mediated pathways may be central to melatonin function, a possibility that has not been sufficiently addressed in invertebrate research.

## Supporting Information

Figure S1 **Representative images of negative controls for melatonin immuno-staining**. (A–D) Negligible fluorescence occurred in whole mounts or sliced tissues when the application of primary antibody was excluded or when the primary antibody was preadsorbed with melatonin (10^−3^ M). (E–H) autofluorescence/non-specific staining in representative control confocal sections. Interference from background or unspecific fluorescence was extremely weak in tissue slices, and the same exposure levels which in stained tissue sections revealed specific and bright fluorescence signals, showed no fluorescence in controls that were subjected to the same immunostaining procedures while excluding the application of primary antibody or when preadsorbed with melatonin (10^−3^ M).
(TIF)

Figure S2 **Representative controls for ISH analyses**. Excluding the application of a labeled probe, using a complementary probe (‘sense’), or excluding the application of the anti-Dig/AP antibody resulted in no significant staining. (A, B) representative control images for putative *HIOMT* orthologs (protein ID’s 91623, 123672). (C, D) representative control images for putative *Nematostella* melatonin receptors orthologs (protein ID’s 209463, 13917).
(TIF)
