# Supplementary figures and images for: Melatonin Distribution Reveals Clues to Its Biological Significance in Basal Metazoans
Source: PLoS One. 2012 Dec 26;7(12):e52266. doi: 10.1371/journal.pone.0052266 (PMC3530593; doi:10.1371/journal.pone.0052266)

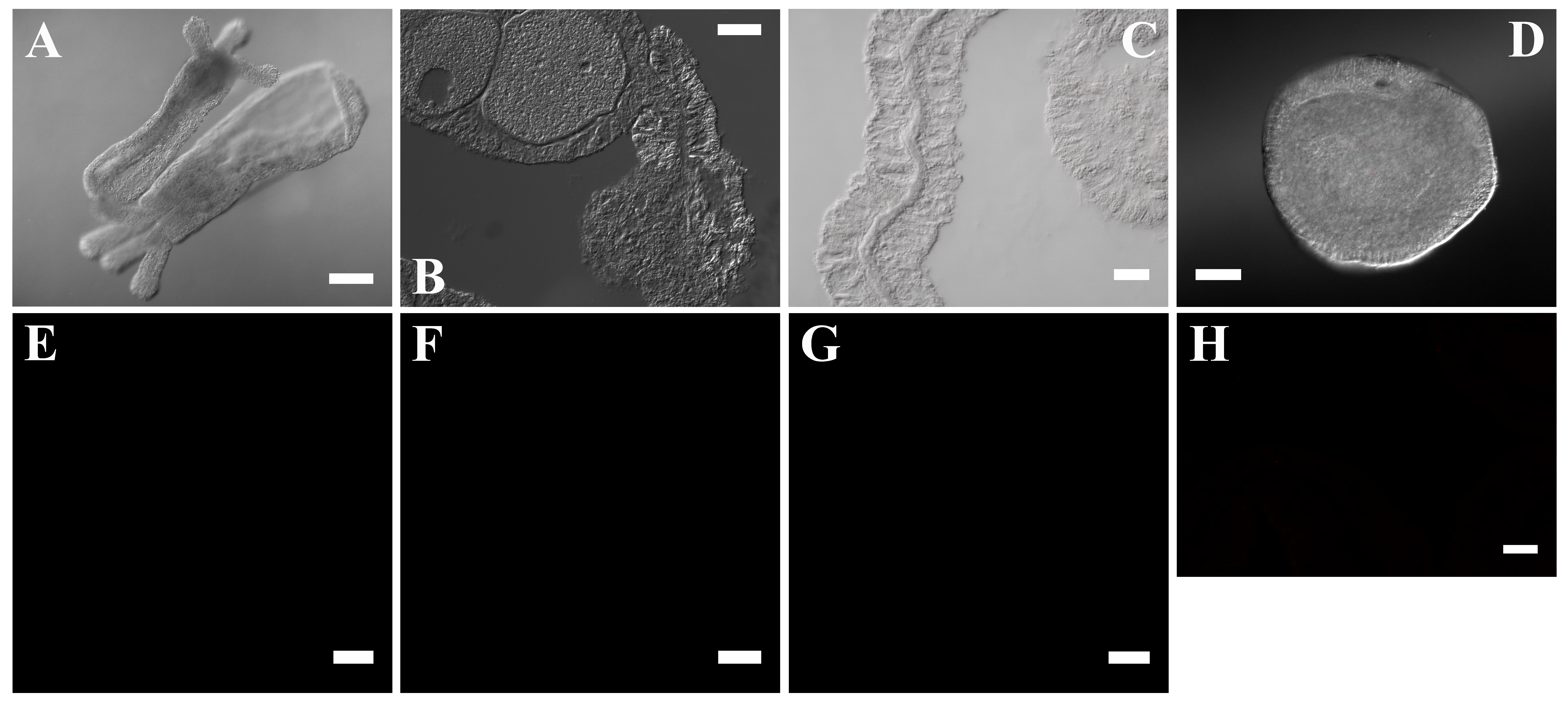

Supplement: Figure S1 — Representative images of negative controls for melatonin immuno-staining. (A–D) Negligible fluorescence occurred in whole mounts or sliced tissues when the application of primary antibody was excluded or when the primary antibody was preadsorbed with melatonin (10−3 M). (E–H) autofluorescence/non-specific staining in representative control confocal sections. Interference from background or unspecific fluorescence was extremely weak in tissue slices, and the same exposure levels which in stained tissue sections revealed specific and bright fluorescence signals, showed no fluorescence in controls that were subjected to the same immunostaining procedures while excluding the application of primary antibody or when preadsorbed with melatonin (10−3 M). (TIF) [file pone.0052266.s001.tif]

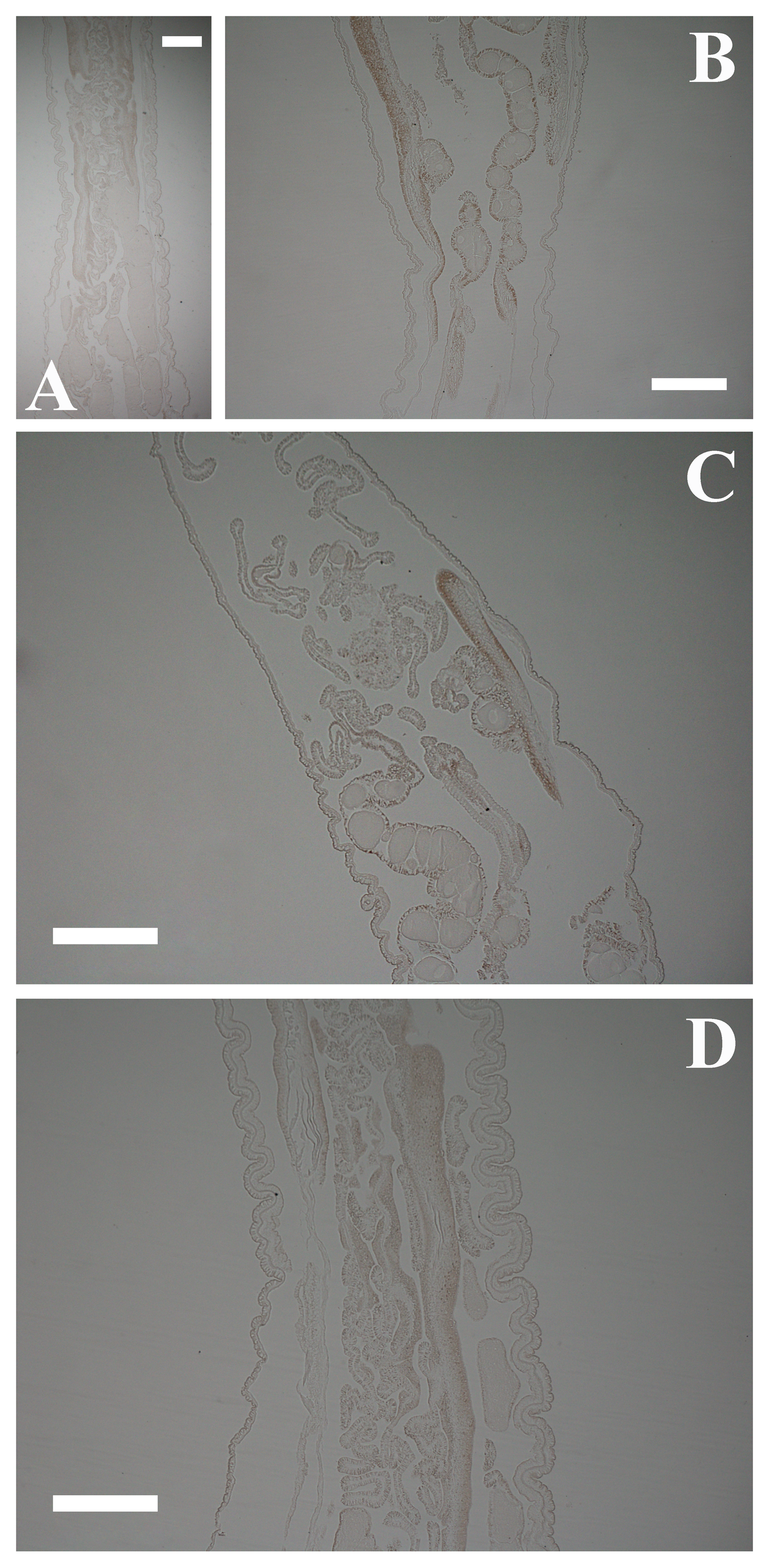

Supplement: Figure S2 — Representative controls for ISH analyses. Excluding the application of a labeled probe, using a complementary probe (‘sense’), or excluding the application of the anti-Dig/AP antibody resulted in no significant staining. (A, B) representative control images for putative HIOMT orthologs (protein ID’s 91623, 123672). (C, D) representative control images for putative Nematostella melatonin receptors orthologs (protein ID’s 209463, 13917). (TIF) [file pone.0052266.s002.tif]
